# Volatile Fingerprinting and Sensory Profiles of Coffee Cascara Teas Produced in Latin American Countries

**DOI:** 10.3390/foods11193144

**Published:** 2022-10-10

**Authors:** Juliana DePaula, Sara C. Cunha, Adriano Cruz, Amanda L. Sales, Ildi Revi, José Fernandes, Isabel M. P. L. V. O. Ferreira, Marco A. L. Miguel, Adriana Farah

**Affiliations:** 1 Laboratório de Química e Bioatividade de Alimentos & Núcleo de Pesquisa em Café Professor Luiz Carlos Trugo—NuPeCafé, Instituto de Nutrição Josué de Castro, Universidade Federal do Rio de Janeiro, Rio de Janeiro 21941-902, Brazil; 2 LAQV/REQUIMTE, Laboratório de Bromatologia e Hidrologia, Departamento de Ciências Químicas, Faculdade de Farmácia da Universidade do Porto, 4099-030 Porto, Portugal; 3 Instituto Federal de Educação, Ciência e Tecnologia do Rio de Janeiro, Rio de Janeiro 20260-100, Brazil; 4 Purity Coffee, Greenville, SC 29609, USA; 5 Laboratório de Microbiologia de Alimentos, Instituto de Microbiologia Paulo de Góes, Universidade Federal do Rio de Janeiro, Rio de Janeiro 21941-902, Brazil

**Keywords:** coffee cherry tea, coffee husk, volatile compounds, coffee fruit, infusion, fermentation

## Abstract

Coffee is one of the most produced and consumed food products worldwide. Its production generates a large amount of byproducts with bioactive potential, like the fruit skin and pulp, popularly called cascara. This study aimed to evaluate the volatile and sensory profiles and the consumption potential of commercial *Coffea arabica* cascara teas by Rio de Janeiro consumers. Analyses of volatile organic compounds in unfermented (*n* = 2) and fermented (*n* = 4) cascara tea infusions were performed by GC-MS. RATA and acceptance sensory tests were performed with untrained assessors (*n* = 100). Fifty-three volatile organic compounds distributed in 9 classes were identified in different samples. Aldehydes, acids, alcohols, esters, and ketones prevailed in order of abundance. With mild intensity, the most cited aroma and flavor attributes were sweet, herbal, woody, prune, fruity, honey, toasted maté and black tea for unfermented teas. For the fermented teas, sweet, woody, black tea, prune, herbal, citric, fruity, honey, raisin, peach, toasted maté, tamarind, and hibiscus were rated as intense. A good association between the attributes selected by the assessors and the volatile compounds was observed. Unfermented teas, with a mild flavor and traditional characteristics, showed better mean acceptance (6.0–5.9 points) when compared to fermented teas (6.0–5.3 points), with exotic and complex attributes. These were well accepted (>8.0 points) by only about 20% of the assessors, a niche of consumers that appreciate gourmet foods.

## 1. Introduction

In response to the growing demand from the consumer market, world coffee production is constantly expanding, having surpassed 10 million tons in 2021, from which approximately 60% corresponded to the production of *Coffea arabica* (arabica coffee) and 40% of *Coffeacanephora* (robusta coffee) [1].

**Figure 1:**
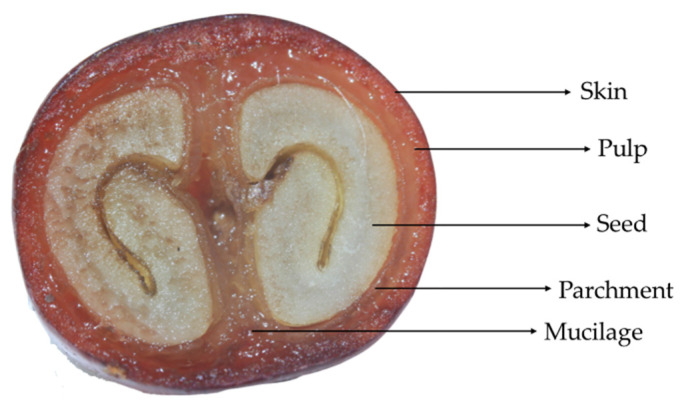
Transverse cut of a *Coffea arabica* fruit, reproduced with permission from the Royal Society of Chemistry.

Several steps are involved in coffee production. After the fruits are harvested, they may undergo different types of processing to release the seeds that are traditionally roasted and ground for the coffee beverage extraction. While in the wet postharvest processing the skin and pulp are fermented or enzymatically digested to release the seeds, in the dry and semi-dry processing, they are mechanically separated from the seeds after washing and drying [2]. The coffee pulp alone corresponds to approximately 28% of the coffee fruit on a dry weight basis, the skin approximately 12%, and the seeds 50–55% [3] (Figure 1).

Tons of this byproduct are generated from coffee fruit processing and discarded annually. It has been estimated that for every million 60 kg bags of dried coffee seeds, about 218,400 tons of dried coffee cherry skin and pulp (the hull or cascara) are generated [4]. When improperly discarded, these residues may negatively impact the ecosystem [5], especially the freshwaters where byproducts from coffee processing usually are drained [6]. Looking from another perspective, these byproducts are rich in bioactive compounds [7] and represent a significant resource that can improve the quality of living of small coffee producers [8,9,10,11]. In this sense, alternatives are proposed to reuse these byproducts in the food industry, for example, extraction of anthocyanins for food coloring [12] and production of eatable flours [13] and tea [8]. In fact, for centuries, the cascara has been used to prepare medicinal infusions in producing countries, where they received different names. Examples are “hashara” in Ethiopia, “qishr” in Yemen, “sultana” in Bolivia, and “cascara” in El Salvador, Colombia [11], and in most parts of the world where it is marketed.

In recent years, controlled fermentation of varying intensities has been carried out during the postharvest processing of coffee fruits. In the process, the whole fruit is fermented, sometimes with the addition of different types of yeast, which resembles grape processing for wine production. Such fermentation results in rich aromatic, fruity and sweet notes, which impregnate the seeds and remaining parts of the fruit and add value to them [2].

In 2017, the European Union interrupted the marketing of *C. arabica* cascara tea because it was considered a “Novel Food”—that had not been consumed to a significant degree in the European Union before 1997—and therefore needed authorization [14]. For this, scientific information involving the determination of chemical composition, microbiological and toxin screening, and safety assessment that proved that people who had previously consumed the product have not developed health problems was necessary [14]. In 2021, considering the nature of coffee cascara and the history of its use as food, the EFSA Panel on Nutrition [15] considered that no more toxicological studies were required, and the risk of allergic reactions was low. The Panel concluded that *C. arabica* cascara was a safe ingredient for preparing non-alcoholic drinks and infusions. Despite this validation as a safe food ingredient, consumption is still overlooked in the West, and very few studies on chemical characterization have been published, especially on its volatile composition. Nevertheless, the considerable content of bioactive compounds and the variable sensory notes make it promising food ingredient or product, providing that it is produced under the health regulatory agencies.

This study evaluated the volatile and sensory profiles of infusions of *C. arabica* cascara teas produced in Latin American countries.

## 2. Materials and Methods

### 2.1. Samples and Study Design

Seven samples of arabica coffee cascara tea were acquired directly from producers. Four originated from wet processed and fermented fruits and three from dehulling from dry/semi-dry processed fruits [2] (Table 1). Samples were ground to pass through an 850 µm sieve for chemical and microbiological analyses. Infusions were prepared using the cascaras as bulk. All samples underwent preliminary microbiological analyses. Based on these results, six were selected for volatile and sensory tests with untrained assessors. Instrumental color, soluble solids, pH, and titratable acidity were determined as complementary analyses.

### 2.2. Infusions Preparation

Infusions were prepared under the food safety requirements of the Brazilian Health Surveillance Agency (ANVISA) [16] and the Food and Agriculture Organization of the US (FAO)/World Health Organization (WHO) [17]. After preliminary sensory tests to adjust the amount of tea and water, 1 L of 90 °C spring water (pH 5.8; bicarbonate: 7.53 mg/L; potassium: 1.669 mg/L; sodium: 1.212 mg/L; nitrate: 4.53 mg/L; chloride: 0.58 mg/L; calcium: 1.402 mg/L; magnesium: 0.585 mg/L; barium: 0.065 mg/L; sulphate: 0.06 mg/L; fluoride: 0.04 mg/L) was poured over 35 g cascara and let steep for 5 min before removing them with a traditional sieve for the preparation of bulk infusions. Samples of the infusions were collected for microbiological, chemical, and physical analyses. The remaining amount was then placed in thermoses for sensory analyses, where it was kept for up to 20 min to ensure a temperature of 68 °C ± 2 °C [18,19,20].

### 2.3. Physical-Chemical Analyses

A portable colorimeter (Konica Minolta, CR-410, Tokyo, Japan) was used to determine the instrumental color of the infusions by the L* (lightness), C* (chroma), *H*° (hue angle) system [21]. Soluble solids were determined in the infusions using the Atago^®^ digital refractometer (model PAL-1, Tokyo, Japan), and results were expressed in °Brix. pH was evaluated using a pH meter (Macherey-Nagel^®^, Düren, Nordrhein-Westfalen, Germany); titratable acidity was determined by titration with 0.1 N NaOH, using phenolphthalein as an indicator, according to Adolfo Lutz Institute [22]. Results were expressed in mEq NaOH/L of infusion.

### 2.4. Analyses of Volatile Organic Compounds

Extraction of the volatile organic compounds from the infusions was performed by HS-SPME, using a 50/30 μm divinylbenzene/carboxen/polydimethylsiloxane fiber (DVB/CAR/PDMS, Supelco^®^), and analyzed by a gas chromatographer (Agilent, 6890 Little Falls, DE, USA) coupled to a mass spectrometer (Agilent 5975) (GC-MS), according to the methodology described by Wang et al. [23]. Before use, the fiber was conditioned according to the manufacturer’s recommendations. 2 mL of each sample were placed in a 20 mL SPME vial, which was immediately sealed with silicone septa and conditioned for 5 min at 50 °C under continuous agitation. Then, the fiber was exposed to the vial headspace for 30 min, in agitation, and heated at 50 °C. After this period, the fiber was retracted, and inserted into the chromatographic injector, in splitless mode, for 2 min, for desorption of volatile compounds, with the aid of a carrier gas (helium) and transferred directly into the chromatographic column (SPB-5, 60 m × 0.32 mm, film thickness df = 1 μm 5%diphenyl—95%dimethylpolysiloxane, Supelco, Bellefonte, PA, USA) at 1 mL min/for 10 min, 250 °C. The chromatographic separation conditions were: 40 °C for 3 min, ramped to 200 °C at 5 °C/min, subsequently ramped to 250 °C at 10 °C/min, and held at final temperature for 3 min. The transfer line, ion source, and MS quadrupole temperature were 250, 230, and 150 °C, respectively. Electron impact mass spectra were measured at the acceleration energy of 70 eV. Data acquisition was performed in full-scan mode from *m*/*z* 50 to 550. Analytes were tentatively identified by the linear retention indices (LRI) and confirmed by the National Institute of Standards and Technology (ChemdataNIST V2.2, Gaithersburg, MD, USA) library database [24]. Agilent Chem Station (Agilent Technologies, Santa Clara, CA, USA) was used for data collection and processing. In addition, high purity external standards, when available, were injected. The LRI of each compound was calculated using the respective retention time (RT) compared against the RTs of a series of standard n-alkanes. The compounds were identified based on their LRI, the mass spectra of the NIST library [24], or authentic standards measured under the same conditions. To identify the compounds, substances with a probability greater than 50% were selected. To improve the accuracy of compounds’ identification, only those substances that provided a match factor higher than 600 and a match factor versus reversed match factor ratio greater than 0.8 were selected for data processing [25]. LRI available from previous publications were also used for comparison.

### 2.5. Microbiological Analyses for Food Safety

Before sensory tests, microbiological analyses for molds and yeasts, total and thermotolerant coliforms, and *Salmonella* spp. were carried out in duplicate, following the standards established by ANVISA [26] and EFSA [15]. Samples (25 mL) were diluted in a culture medium (BHI broth—Brain Heart Infusion, Difco) and homogenized (Stomacher—Splabor^®^, SP-190) before the analyses. *Escherichia coli* ATCC (American type culture collection) 25922 and *Salmonella enteritidis* ATCC 13076 were used as control strains.

### 2.6. Sensory Analysis

The sensory tests were previously approved by the Research Ethics Committee of Clementino Fraga Filho University Hospital (# 21776619.8.0000.5257). One hundred assessors, who consumed plant infusions habitually, took part in the acceptance, purchasing intention, and Rate All That Apply (RATA) tests. They were students, teachers, visitors, and employees at the Federal University of Rio de Janeiro-UFRJ Health Sciences Center and Technology Center. After being informed of the procedures and expressing their agreement, they signed the Informed Consent Form. Assessors performed the tests on individual benches in the UFRJ Food and Dietetics Lab under white light. Before receiving the samples, demographic information was collected, including gender, age, educational level, occupation, family monthly income, frequency, and habits of infusions consumption. Approximately 30 mL of each infusion were presented at a time in 50 mL polystyrene plastic cups, coded with three-digit random numbers, and distributed in a balanced way to avoid the consistent influence of neighboring samples on the sensory sensation. Assessors were instructed to drink the infusions with or without sweeteners as they habitually did. They could use table sugar or artificial sweetener (saccharin or aspartame). They were advised (and monitored) to use the same type and amount of sweetener in all samples. Crackers and spring water at room temperature were offered between samples to clean the palate.

#### 2.6.1. Consumer Acceptance and Purchase Intention

Assessors evaluated the infusions using the nine-point hedonic scale ranging from 1 (extremely disliked) to 9 (extremely liked), followed by a five-point hedonic scale ranging from 1 (certainly would not buy) to 5 (certainly would buy) [27]. The Acceptability Index (AI) was calculated using the following equation: AI = (X × 100)/N, where: X = Average score given by assessors and N = Highest score given by assessors. AI equal to or greater than 70% was considered satisfactory [27].

#### 2.6.2. Rate All that Apply (RATA)

After marking the hedonic scales, assessors were given a pre-prepared checklist with 30 sensory attributes related to appearance, aroma, flavor, and mouth feel, which were identified in the preliminary session performed by the trained panel. Following, they were required to select all terms they considered appropriate to describe the infusions. Given that fermented coffee cascara infusions had a more pronounced aroma, flavor, and taste attributes than the unfermented ones, we investigated whether the assessors perceived such differences by asking them to score the attributes according to their intensity (RATA scores: 0 = no perception; 1 = low intensity; and 2 = high intensity) [28,29]. The attributes used in the study were organized by alphabetical order as follows: acidic, apricot, astringent, bitter, black tea, brown, burnt, citric, fermented, floral, fruity, green coffee, green leaf, herbal, hibiscus, honey, jasmine, liquor, mouthfeel, orange-brown, toasted maté, peach, prune, raisin, sweet, tamarind, toasted leaf, tobacco, wine, and woody.

### 2.7. Statistical Analysis

Data from physical and chemical analyses were analyzed using GraphPad Prism (Version 8.4.2, Informer Technologies, Los Angeles, CA, USA) and presented as mean ± standard deviation. They were compared for differences by one-way ANOVA, followed by the Tukey test, at a 5% significance level. Pearson’s correlation was used to compare soluble solids and color parameters. Data from sensory tests were analyzed using XLSAT for Windows (Version 2019.3, Boston, MA, USA). ANOVA was used for acceptance and purchase intention tests, with two sources of variance and a distribution histogram, considering the socio-demographic and consumption data obtained through questions. Cluster analysis based on the hierarchical grouping of acceptance scores was carried out to identify segments of consumers with similar likings.

RATA scores were analyzed as continuous data, with Principal Component Analysis (PCA), through the arithmetic mean values of the sensory descriptors for all assessors. Non-applicable attributes were marked as intensity 0 [28,29]; the mean overall liking scores and the quantitative analysis of each organic function detected on the volatile profiling were considered as supplementary. 95% confidence ellipse generated by virtual panels using Bootstrap techniques were used. For each consumer panel, linear mixed-effects models were performed for overall liking and sensory attribute intensities to examine if there were significant differences across the samples, with the sample as a fixed effect and the assessor as a random effect. Tukey’s HSD test was used for post-hoc pairwise comparisons of sample means [30].

## 3. Results and Discussion

### 3.1. Microbiological Analysis

Among the seven analyzed samples, one (sample 7) contained the number of total coliforms (2.4 × 10^4^ Most Probable Number—MPN/g) and thermotolerant coliforms at 45 °C (2.5 × 10^4^ MPN/g) above the limit allowed by the Brazilian (10^2^ MPN/g) [26] and European [15] legislations. Escherichia coli and *Salmonella* sp., molds, and yeasts (4.6 × 10^3^ Colony Forming Unit—CFU/g) were also identified in this sample. Therefore, it was excluded from the study.

Coliforms, including *E. coli* and *Salmonella* spp., are among the etiological agents frequently identified in outbreaks of foodborne diseases [31]. The count of total coliforms and *E. coli* reflects the current health condition of the food [32] and indicates fecal contamination [33]. The counting of yeasts and molds in foods can also be used to assess the quality and progression of deterioration and predict the product’s shelf life [34]. However, in some cases, like intentional fermentation during postharvest processing of coffee cherries, yeasts can be used for flavor development. Consequently, fermentation must be carried out in a controlled manner to avoid the growth of undesirable microorganisms.

Because hot water is used for tea preparation, all the infusions, including the one from Sample 7, were microbiologically safe, with zero counts of thermo-tolerant coliforms at 45 °C, *E. coli*, *Salmonella* sp. and molds and yeasts. According to the Food and Agriculture Organization [35], the limit of viability and multiplication of pathogenic strains of *E. coli* in foods is between 6.5–49.4 °C. Despite the death of yeasts and molds, a concern is the susceptibility of coffee cascara to contamination by mycotoxin-producing microorganisms [36]. Ochratoxin A (OTA) is the most studied mycotoxin in coffee and the only one regulated by European legislation [37]. A recent study has detected OTA (4.3 μg/kg) in a coffee cascara tea from Nicaragua at a lower level than that established by the EC (maximum 5 μg/kg) [13]. Hot water cannot destroy mycotoxins, which are reasonably soluble [38]. Therefore, coffee cascara may present food security risks common to those observed in dried fruits. Considering the microbiological results, Sample 7 was excluded from the remaining parts of the study. It is worth noting that in the case of iced tea preparation, coffee cascara should always be extracted with hot water and then refrigerated, never cold brewed.

### 3.2. Physical-Chemical Analyses

**Figure 2:**
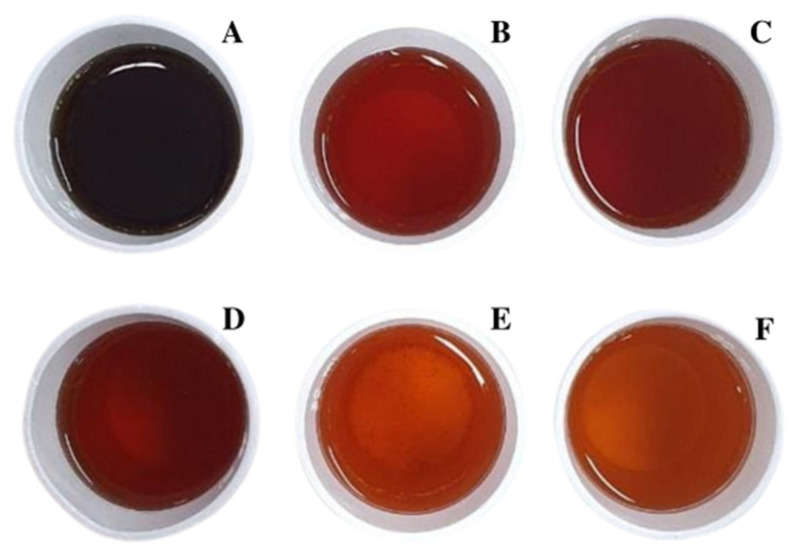
Infusions of commercial coffee cascara teas from Latin American countries. (**A**): sample 3—El Salvador (F); (**B**): sample 1—Brazil (F); (**C**): sample 4—Nicaragua (F); (**D**): sample 5—Brazil (UF); (**E**): sample 6—Nicaragua (UF); (**F**): sample 2—Bolivia (F). Note: F–fermented; UF—unfermented.

Figure 2 shows the visual appearance of the coffee cascara infusions evaluated in this study. Table 2 contains the instrumental soluble solids, color pH, and titratable acidity data.

Soluble solids values (range 1.10–1.30 °Brix, using 3.5 g cascara/100 mL) were higher in Samples 3 and 4 (both fermented). The present results are higher than those previously reported for arabica coffee cascara infusion (0.54) prepared using 1 g cascara/100 mL water [39], bulk black tea (0.45) and bulk maté tea (0.23) (both using 1.6 g/100 mL) [40].

The colorimetric data varied for the different infusions. L* represents the lightness measured as brightness, with 100 and 0 values corresponding to absolute white and black, respectively [41]. The L* values of infusions were found to be significantly lower (darker) in Sample 3 (56.7 ± 1.5) and higher (lighter) in Sample 2 (79.7 ± 0.5) (both fermented) compared to the other samples, as visually perceived in Figure 2. C* represents the chroma or intensity or saturation, the degree of color relative to a similarly illuminated neutral grey [41]. C* values were the highest in Samples 4, 1 and 3. This indicates that these fermented samples had more vivid colors. *H°* represents the hue [41]. *H*° values of fermented infusions varied between 76.71° and 97.58°, while the values of unfermented infusions were 90.96° and 96.03°. The lower *H*° value in Sample 3 indicates that the infusion was more reddish and less yellowish, and the higher *H*° value in Sample 2 indicates the infusion was more yellowish. Although the fermented cascaras were darker than the unfermented ones (exemplified in Table 1), this was not necessarily reflected in the infusions. In Figure 2, the infusions in cups A and F were from fermented cascaras. However, while cup A presented the highest soluble solids value (1.30 °Brix), cup F presented one of the lowest values (1.13 °Brix) (Table 2). Nevertheless, positive correlations were observed between soluble solids and L* (r = 0.7908, *p* = 0.014), C* (r = 0.6757, *p* = 0.047), and *H*° (r = 0.8549, *p* = 0.0083). The variety and intensity of colors observed in the infusions are related to the presence of anthocyanins, responsible for the red, yellow, and purple colors observed in coffee fruit skin, mainly cyanidin-3-rutinoside, and, in small amounts, cyanidin 3-glycoside [12].

In the present study, pH and TA values were generally similar in all infusions. The pH values (ranged 4.18–4.22) are relatively similar to those previously reported for arabica cascara infusions prepared using 2 g cascara/100 mL (range 4.55–4.62) [42] and lower than those prepared using 1 g cascara/100 mL [39], confirming the acidic characteristics of cascara coffee tea and showing that the pH values are related to the amount of cascara used to prepare the infusions.

### 3.3. Volatile Organic Compounds

Considering all infusions, 53 compounds (corresponding to 89.3–97.1% of the total peak areas of the chromatograms) were accurately identified (Table 3). They were grouped into 9 chemical classes: 16 aldehydes, 9 acids, 8 esters, 6 alcohols, 4 terpenoids, 3 ketones, 3 furans, 2 pyrroles and 2 pyrazines. Of the 53 compounds, only 6 were present in all samples, 9 in both unfermented and 13 in the four fermented samples. The remaining compounds were differently distributed among samples, which differed not only concerning the number of compounds but also in chemical classes (especially aldehydes, acids, alcohols, and ketones) and peak areas. Most identified compounds, a few reported as odoriferous components of other foods, were generated by fermentation, which is a key step in aroma development [43].

Despite the significant variation in the volatile profiles among samples, most identified compounds have been recently reported by Pua et al. [44] who have examined infusions from five coffee cascara teas, also from different countries. A few compounds reported by these authors have been detected in this study but did not meet the peaks confirmation criteria; therefore, they were not considered. No other study investigating the volatile composition of coffee cascara infusions was found. Two studies have investigated the volatile profile of fresh *C. arabica* [45] and *C. canephora* [46] pulps. Of the 45 and 55 volatile compounds reported in these studies, only 4 and 10 were identified in the present study, respectively, suggesting that the volatile profile of coffee cascara changes considerably as the fruit is fermented and or dried.

Aldehydes represented about 19.5–39.0% of the total peak areas, with higher percentages in the infusions from fermented teas, especially Sample 2. Aldehydes contribute remarkably to citrus, fruity, floral, fresh, and herbaceous notes [55]. Some of these compounds, such as octanal, decanal and dodecanal, identified in this study, have been listed as impact compounds in citrus fruits and have attractive sensory qualities, according to aroma and flavor assessments [49,50]. Octanal has also been listed as one of the main aroma-active compounds responsible for the unique aroma of toasted maté leaf [51]. Hexanal, heptanal, nonanal and benzaldehyde have been listed as key aroma and flavor compounds in black tea [23,52]. Hexanal has been previously identified in fresh *C. arabica* [45] and *C. canephora* [46] cherry pulps. Nonanal, which also has been identified in fresh *C. canephora* cherry pulp [46], contributes to citrus, fresh, orange, and green characteristics [53,54]. This aldehyde was the most relevant concerning the chromatogram peak areas in all evaluated teas, accounting, in the fermented samples, for 9–15% of the total chromatogram peak area and, in the unfermented samples, for 15–20%. Because of their high chemical reactivity, the concentration of aldehydes is significantly altered during thermal processing [56] and, therefore, boiling these teas is probably not recommended.

Acids accounted for 11.0–33.4% of the total chromatogram peak area, with the highest percentages found in infusions from fermented teas, especially Samples 2 and 3. Acids contribute pungent, vinegar, sour, fermented, and acidic notes. Acetic acid, also identified in the fresh *C. canephora* pulp [46], has been listed as a key aroma compound in fermented beverages [51]. Octanoic and nonanoic acids were the most relevant in all samples concerning peak area, except for Sample 5 (unfermented), in which decanoic acid was more relevant. These compounds generally impart acid, sweat, and fruit characteristics to the beverage [53,54].

Alcohols accounted for 3.3–11.4% of the total peak area of the volatile fraction of different samples, with higher percentages in the infusions from fermented teas, especially Sample 2. Phenylethyl alcohol, identified in fresh *C. canephora* pulp [46], has been reported as key aroma compound in black tea [23,52]. Together with ethyl hexanol, it was the most relevant alcohol in all samples. Alcohols, in general, contribute honey, floral, fresh, rose, citrus and alcohol notes [53,54].

Esters comprised 3.0–23.5% of the total peak area of the chromatograms. The highest percentages of esters were observed in the fermented infusions. These compounds are essential volatile components in many fruits, and most of them have a strong fruity and floral odor and contribute to the “mature” flavor [57,58]. Methyl and ethyl salicylate have been previously identified in fresh pulps of *C. arabica* [45,59] and *C. canephora* [46]. Methyl salicylate has also been identified in Pu-erh tea samples and reported as an important component for the overall tea aroma formation [60].

Terpenoids comprised 2.8–8.5% of the total peak area of the chromatograms. Although these compounds are characterized by poor aroma, they still impart sweet, citrus, fruity, woody, and herbal characteristics [55]. However, they are susceptible to degradation reactions when exposed to air (oxygen), light, heat and undergo consequent conversion to terpenic alcohols or oxides [55]. Linalool, the most relevant terpenoid in the cascara teas, has been identified in fresh *C. arabica* [45] and *C. canephora* [46] pulps. It has been reported as key aroma compound in black tea [23,50,52]. β-ionone, only identified in Sample 2 (fermented), has also been listed as one of the main aroma-active compounds in toasted maté leaf [49].

Ketones comprised 3.0–13.3% of the total peak area of the volatile fraction of the different teas, with the highest proportion found in infusions from fermented samples, especially Samples 1, 2 and 3. Biogenetically, ketone components are derived from alcohols through oxidation reactions catalyzed by different enzymes, which exert dehydrogenase activity, and have a relevant contribution to the taste and fragrance of the essential citrus fruit oils [56]. In general, they contribute sweet, fruity, rose, and honey notes [53,54]. γ-nonalactone was only identified in Sample 5 (fermented). β-damascenone, identified in the present study only in fermented teas, has been reported as key aroma compound in black tea [52], toasted maté leaf [49] and fruits, vegetables, and derived products, including wine, where it imparts a pleasant “stewed apple”, “fruity” and honey-like character [61]. β-damascenone and γ-nonalactone have been cited as compounds responsible for prune aroma in prematurely aged red wines [62]. β-damascenone has also been previously identified in fresh robusta cherry pulp [46].

Pyrazines and furans, especially 5-methyl-furfural, are heat treatment markers in dried fruits [44]. Lactones such as γ-nonalactone, are also typical of dried fruits like apricots [63]. Their presence in coffee cascara suggests the importance of the drying process for their overall aroma profile. Pyrazines (2,3,5 trimethyl-pyrazine and 2,6-dimethyl-pyrazine), which contribute roast, nut, cocoa, and caramel notes [53,54], were only identified in one fermented sample. Furfural was identified in all samples, while 5-methyl-furfural and 2-acetyl-furan were only identified in one fermented sample, giving bread, almond, sweet and woody characters [53,54].

In general, alcohols, esters, aldehydes, acids, and ketones have been found to be predominant in dried fruits like raisins [64] and apricots [63]. Overall, the diverse volatile compounds identified in the cascaras reflected their nature as dried fruits [44].

A few alkanes were observed in the infusions, but they were not considered because they did not meet the peaks confirmation criteria. In general, these compounds have a limited effect on the overall odor characteristics of teas [23]. Additionally, small peaks of non-volatile compounds, such as caffeine, lycoxanthin, lycopene and astaxanthin, were identified most probably due to sublimation caused by heat exposure during volatile analysis.

### 3.4. Sensory Tests

As far as the authors know, this is the first study reporting the characterization, acceptance, and purchase intention evaluation of coffee cascara infusions by consumers.

#### 3.4.1. Assessors’ Characteristics

The study assessors’ main characteristics are presented in Table 4. Although the high participation of females in the study can be attributed to their higher willingness to take part in it, it is worth mentioning that women are now responsible for purchasing decisions in most Brazilian homes, and they drink more tea (51.6 mL/day) than men (45.0 mL/day) [65]. Moreover, women around the world traditionally consume more tea and herbal infusions than men, although, with the world’s increase in the availability and consumption of exotic and gourmet products, men tend to consume more herbal infusions increasingly [66].

#### 3.4.2. Consumer Acceptance and Purchase Intention Test Scores

**Figure 3:**
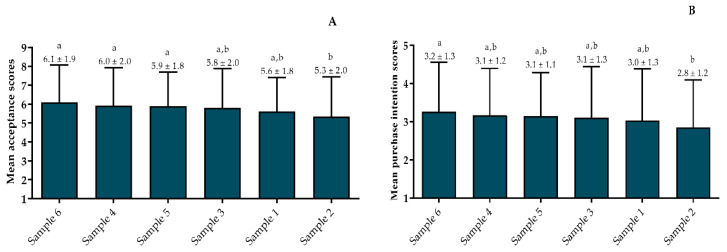
Mean acceptance (**A**) and purchase intention (**B**) scores given for coffee cascara infusions by Brazilian consumers living in Rio de Janeiro. Different letters over the bars indicate statistical differences among samples by ANOVA (*p* ≤ 0.05) (*n* = 100 assessors). Samples: 1—Brazil (F); 2—Bolivia (F); 3—El Salvador (F); 4—Nicaragua (F); 5—Brazil (UF); 6—Nicaragua (UF). F—fermented; UF—Unfermented.

The mean acceptance scores for all infusions ranged from 5.3 (nor liked neither disliked) to 6.1 (liked slightly) (Figure 3A). No significant difference was observed between the genders’ scores. The mean Acceptability Index (AI) ranged from 67.8% to 59.4%.

According to Meilgaard et al. [27], for a sample to be considered “well-accepted”, it must obtain 70% AI or higher. Therefore, considering the mean scores from all assessors, the tested infusions failed to reach 70%, although Sample 6 (unfermented infusion) (68%) was close to reaching it. As usual, the purchase intention results (Figure 3B) were associated with those from the acceptance test. Sample 2 (fermented) received a lower score, probably because of the high degree of fermentation.

As individual preferences may differ considerably concerning foods and are not reflected in the mean scores, cluster analysis was carried out to identify consumer segments with similar likings. This analysis is relevant for distinguishing different market niches. Three groups of consumers were identified.

Cluster 1 (*n* = 22, mean score = 8—“liked very much”, and AI ≈ 87%) consistently attributed the highest scores, especially to the unfermented samples. Cluster 1 was primarily composed of females (73%) aged between 25 and 34 years old (64%), with postgraduate education (68%), family monthly income > 5 MW (59%). They habitually consumed caffeinated infusions such as toasted maté (55%) and black tea (32%), and herbal infusions such as lemon balm (64%) and chamomile (59%). They also reported consuming fruity teas, such as hibiscus, lavender, and roses (50%). Most assessors usually consumed imported teas and did not have the habit of sweetening teas.

Cluster 2 (*n* = 30, mean score = 4—“disliked slightly”) was composed mostly of female (66.7%), 18–24 years old (56.7%), with incomplete higher education (60%), and family monthly income of 2 to 3 MW (46.7%). They habitually consumed infusions such as toasted maté (53.3%) and herbal or fruit infusions (40.0%).

Cluster 3 (*n* = 48, mean score = 6—“liked” or “slightly liked”), the largest cluster, was composed mostly of females (81.3%), 18–24 years old (54.2%), with incomplete higher education (47.9%), family monthly income between 2 and 3 MW (47.1%). They habitually consumed infusions of toasted maté (60.4%), chamomile (43.8%) and lemon balm (43.8%).

These findings suggest that post-graduate women aged 25–34 years with family monthly incomes higher than 5 MW are potential consumers of cascara coffee tea. This consumer tends to appreciate exotic and expensive gourmet products [67]. On the other hand, young consumers aged 18–24 years, with lower income and education, are not good candidates.

#### 3.4.3. Rate All that Apply (RATA) Test

**Figure 4:**
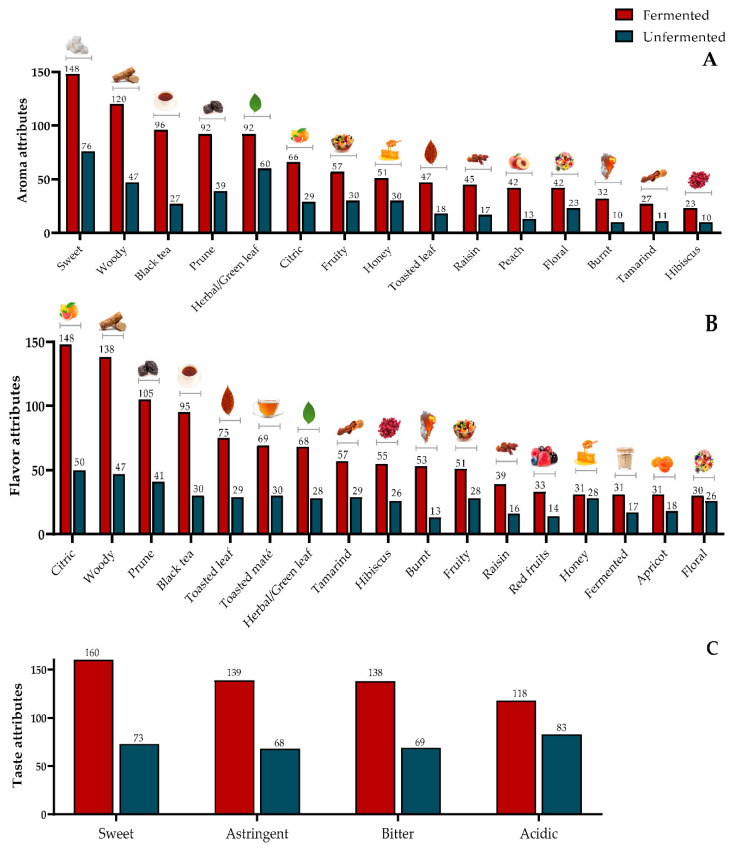
Main aroma (**A**), flavor (**B**) and taste (**C**) attributes selected for all coffee cascara infusions by the assessors.

**Figure 5:**
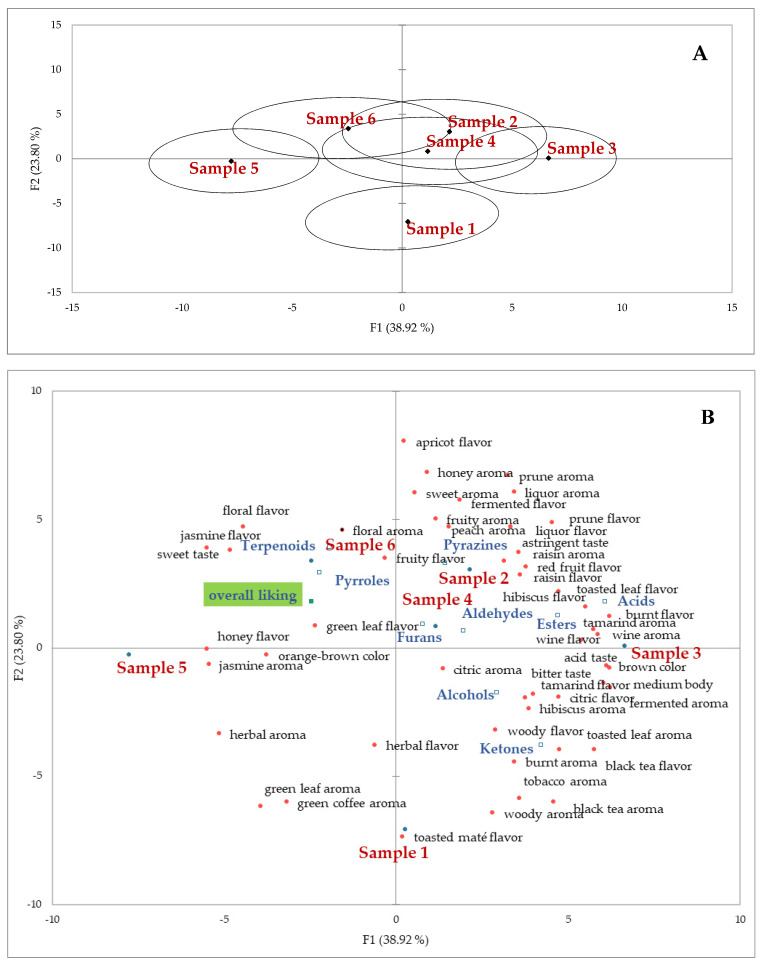
Principal component analysis (PCA): bi-dimensional plot of samples of cascara coffee tea infusions (*n* = 6) (**A**) and sensory characteristics attributed by consumers (*n* = 100) through RATA sensory test, distributing volatile compounds and attributes that make up the best acceptance of samples among consumers (**B**). Overall liking and the volatile compounds were considered as supplementary variables. Samples: 1—Brazilfermented (F); 2—Bolivia (F); 3—El Salvador (F); 4—Nicaragua (F); 5—Brazilunfermented (UF); 6—Nicaragua (UF).

RATA test results are presented in Figure 4 and Figure 5. Considering all samples, Figure 4 presents the main sensory attributes and the number of times the assessors checked them.

Considering the results for each sample individually, on the right side of Figure 5a, the superimposition of the ellipses around samples 2, 3 and 4 (fermented samples) indicate that they present very similar characteristics. Samples 5 and 6 (unfermented samples) are located on the left side of the figure and carry similar characteristics, although Sample 6 exhibited a few attributes similar to the fermented samples; Sample 1 was placed in an intermediate position given its hybrid characteristics (mildly fermented). Figure 5b presents the sensory attributes reported for the individual samples by the assessors in association with the acceptance scores and classes of volatile compounds used as secondary variables. The individual volatile compounds and their characteristic attributes are presented in Table 3.

Looking at Figure 4 and Figure 5, it is notable that, in general, the fermented teas showed more richness of sensory attributes when compared to the unfermented ones, which still maintained some of the characteristics of green coffee seeds. Nevertheless, the attributes leading to higher acceptance (overall liking) were closer to the unfermented samples because most of these assessors are used to consuming unfermented teas with poor (weak) flavor characteristics. Most Brazilians generally do not expect a fermented beverage when consuming teas because fermented attributes often resemble spoiled foods. Even fruity and flowery teas are not very popular among these consumers. Mostly, the niche of consumers that appreciate gourmet foods and look for exotic and complex attributes will appreciate this type of beverage. Similar situations have been observed in our studies with specialty coffee beverages.

It is worth noting that the distribution of chemical classes in Figure 4B only considered the number of volatile compounds in each chemical class, together with the attributes and their RATA scores, but it did not consider the chromatogram peak areas or the odor threshold of the compounds. Nevertheless, the distribution of the classes is reasonably similar to the odor and flavor descriptions in the literature, which can be revisited in Table 3.

The aroma, flavor and taste of coffee cascara tea vary considerably and are not easy to reproduce, as they depend not only on genetics but also on the terroir, edaphoclimatic conditions and the postharvest processing method applied, including fermentation [2]. The perceived intensity and the number of assessors who perceived the attributes also varied among samples. However, considering the number of volatile compounds commonly identified in the unfermented and or fermented samples, that 26 attributes were frequently checked for all samples and that most volatile compounds identified in this study were also reported by Pua et al. [44], it appears that the volatile profiles presented in this study can characterize coffee cascara teas generically. Nonetheless, it is expected that the cascara teas will most often show unique profiles depending on the conditions aforementioned.

For the unfermented infusions, sweet, herbal, woody, prune, fruity, and honey aromas, and citric, woody, prune, toasted maté, and black tea flavors were most cited but with mild intensity. Orange-brown color, sweetness and light body were often perceived in unfermented teas. Aldehydes and linalool, a terpenoid compound, are related to sweet, herbal, and citric attributes, while phenyl-ethanal and phenyl-ethyl alcohol are related to honey notes [53,54]. Hexanal, benzaldehyde and octanoic acid are related to fruit notes and furfural to woody attributes [53,54] (Table 3).

A variety of aromas (sweet, woody, black tea, prune, herbal, citric, fruity, honey, raisin, peach) and flavor (citric, woody, prune, black tea, toasted leaf, toasted maté, herbal, tamarind, hibiscus, burnt) attributes in fermented teas were marked as intense. In addition to the volatile compounds and attributes observed in the unfermented teas, esters, such as ethyl octanoate and ethyl palmitate and ketones, such as β-damascenone, γ-nonalactone, and geranyl acetone are mainly related to intense fruity aroma and flavor [53,54]. These are the chief compounds responsible for the aroma of prune [62], raisin [64,68], and peach [69]. Furans, mainly 5-methyl-furfural and 2-acetyl-furan and pyrazines, are related to toasted and burnt attributes [53,54]. Color attributes were also, in general, more intense in fermented than in unfermented teas. Additional perceived attributes were: acidic, bitter, astringent, and medium body.

The least mentioned attributes were wine, liquor, jasmine, green coffee, and tobacco. This may result either from low concentration and or high odor threshold of the compounds associated with these attributes or from the fact that these assessors do not experience these aromas habitually, given that only those who are used to consuming those foods or have them in their olfactory memory can recognize them. Although only a few assessors marked these attributes, a few compounds which agree with these attributes were identified in the analyses of volatile compounds. For example, 1,2-epoxylinalool, an alcohol compound only identified in fermented samples, contributes alcohol characteristics, while heptanal, an aldehyde identified in Samples 1, 2, 3 (all fermented) and in one unfermented (Sample 6), contributes wine notes. Benzyl alcohol, linalool, and benzyl acetate are key volatile compounds in jasmine tea [70]. Hexanal, benzaldehyde and hexanoic acid, have been reported in green coffee seeds [71]. In addition to impart pleasant characteristics, such as fruity, sweet and honey, β-damascenone may also contribute to unpleasant tobacco notes (Table 3).

As for taste attributes, bioactive compounds from coffee seeds such as caffeine, trigonelline, and a few polyphenols also identified in coffee cascara teas [7] are related to bitterness [2]. Astringency usually involves the association of polyphenols with proteins in the saliva to form precipitates [2]. Like other polyphenols, such as procyanidins and tannins, chlorogenic acids, the main phenolic compounds in coffee cascara tea [7], generate astringency in the mouth, which, depending on the intensity, may contribute negatively to flavor [72]. During processing, fermentation tends to degrade phenolic compounds and other components in coffee mucilage, providing positive changes in flavor development, including a decrease in astringency [73]. The main compounds responsible for acidity in coffee are non-volatile organic acids, but low-molecular-mass organic acids also contribute to acidity and formation of aroma and flavor [2]. Organic acids contribute specific types and intensity of acidity in different ways, depending on the sensory characteristics, concentration in the beverage and their strengths [2]. To date, citric, malic, citramalic and gluconic acids have been identified in coffee pulp [74].

## 4. Concluding Remarks

In this study, coffee cascara teas were characterized in terms of volatile profiles and sensory attributes. The role of fermentation in flavor development is undeniable, just like for coffee seeds, transforming simple and mild flavor attributes into rich, exotic, and intense flavors. However, traditional caffeinated and herbal teas consumers from Rio de Janeiro did not expect such complexity, which affected acceptability. These consumers may need to dilute the infusions to experience more mild flavors. We believe that if adequately processed from a microbiological and sensory point of view and diluted, coffee cascara infusions, especially those using unfermented cascara, with poor flavor characteristics, can be accepted by the general consumers of Rio de Janeiro, as well as by gourmet foods consumers. Particularly, adult women with greater purchasing power and higher education appreciated all cascara teas, including those with intense exotic and fruity flavor. Thus, each tea singularity and individual preferences should be considered when choosing the preparation method.

Just as there is a fast-growing niche of specialty coffee consumers worldwide who appreciate flavor complexity, we expect a similar trend happening with cascara teas. In addition to the pleasure of drinking a flavorful beverage, coffee cascara tea is a source of bioactive compounds and a way of supporting sustainability in coffee production. Nevertheless, the risk of cascara contamination by mycotoxin-producing microorganisms does exist, and we hope farmers and agronomists will improve their methods to produce “clean” cascara teas to be appreciated by a growing number of consumers. The potential presence of soluble pesticides in the cascara is another concern. Despite the higher costs of organic coffee production, supporting consumers’ health and the environment aggregates product value, and therefore, cascara teas from organic coffee production show potential.

## Figures and Tables

**Table 1:** Samples of commercial fermented and unfermented coffee cascara teas produced in Latin America countries.

	Fermented	
	Sample	Origin/Cultivar
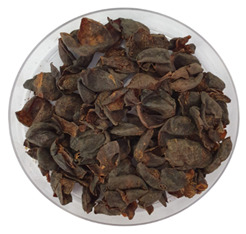 Typical appearance of fermented arabica coffee cascara teas.	**1**	Brazil/Mix of six cultivars: Iapar 59, Bourbon Amarelo, Catucaí Açu, Catuaí 44, Caturra, IcatuPrecoce/Sold in Brazil
	**2**	Bolivia/Nr/Sold in Canada
	**3**	El Salvador/Nr/Sold in the USA
	**4**	Nicaragua/Caturra and Bourbon/Sold in the USA
	**Unfermented**	
	**Sample**	**Origin/Cultivar**
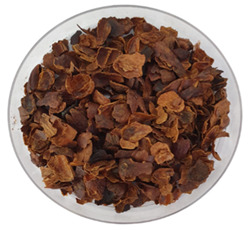 Typical appearance of unfermented arabica coffee cascara teas.	**5**	Brazil/Mix of six cultivars: Iapar 59, Bourbon Amarelo, Catucaí Açu, Catuaí 44, Caturra/Sold in Brazil
	**6**	Nicaragua/Caturra/Sold in the USA
	**7**	Brazil/IcatuPrecoce/Sold in Brazil

Nr: not reported.

**Table 2:** Results from physical-chemical analyses of commercial coffee cascara infusions produced in Latin American countries.

Samples	Instrumental Color			SS (°Brix)	pH	TA (mEq NaOH/L)
	L*	C*	*H*°			
1	70.97 ± 0.86 ^d^	52.11 ± 1.39 ^b^	88.47 ± 0.88 ^d^	1.17 ± 0.06 ^ab^	4.21 ± 0.01 ^a^	6.0 ± 0.3 ^c^
2	79.69 ± 0.50 ^a^	32.54 ± 1.50 ^d^	97.58 ± 0.70 ^a^	1.13 ± 0.06 ^c^	4.22 ± 0.01 ^a^	6.5 ± 0.0 ^b^
3	56.69 ± 1.50 ^f^	51.42 ± 0.23 ^b^	76.71 ± 1.61 ^f^	1.30 ± 0.00 ^a^	4.20 ± 0.00 ^a^	6.5 ± 0.1 ^b^
4	68.98 ± 1.19 ^e^	58.01 ± 1.46 ^a^	85.56 ± 1.19 ^e^	1.27 ± 0.06 ^a^	4.19 ± 0.01 ^ab^	7.2 ± 0.3 ^a^
5	73.68 ± 0.45 ^c^	45.75 ± 0.92 ^c^	90.96 ± 0.49 ^c^	1.20 ± 0.00 ^ab^	4.18 ± 0.01 ^b^	6.2 ± 0.3 ^bc^
6	77.89 ± 0.69 ^b^	34.27 ± 1.40 ^d^	96.03 ± 0.38 ^b^	1.10 ± 0.00 ^c^	4.22 ± 0.02 ^a^	7.0 ± 0.4 ^ab^

Note: L* (lightness), C* (chroma), *H*° (hue angle); SS (soluble solids); TA (titratable acidity). Different letters over the bars indicate statistical differences among samples by ANOVA (*p*≤ 0.05). Samples 1—Brazil (F); 2—Bolivia (F); 3—El Salvador (F); 4—Nicaragua (F); 5—Brazil (UF); 6—Nicaragua (UF). F—fermented; UF—unfermented.

**Table 3:** Volatile compounds identified in infusions of fermented and unfermented commercial coffee cascara teas from Latin American countries and their classical odor description.

					*Samples*					
					Fermented				Unfermented	
Volatile Compound	Odor Description	^a^CAS#	^b^ELRI	^c^LRI	*1*	*2*	*3*	*4*	*5*	*6*
**Aldehydes**										
Pentanal	Almond, pungent, coffee,chocolate [47,48]	110-62-3	749	873	□	■^d,e^	□	□	□	□
Hexanal *	Green, vegetable, fruity, tallow, fat [47,48]	66-25-1	759	874	■^e^	■^d,e^	■^d,e^	■^d,e^	□	■^e^
Hexenal	Apple, green [47]	505-57-7	892	920	□	■^e^	□	□	□	□
Heptanal *	Fresh, herbal, fatty, citrus, wine-lees [47,48]	111-71-7	785	843	■^e^	■^e^	■^d,e^	□	□	■^e^
Octanal *	Citrus, soap, lemon, herbal, honey [47,48]	124-13-0	726	805	■^e^	■^e^	□	■^e^	□	■^e^
Nonanal *	Fat, citrus, fresh, orange, green [47,48]	124-19-6	798	880	■^e^	■^d,e^	■^d,e^	■^e^	■^d,e^	■^d,e^
Decanal *	Sweet, citrus, floral, soap, orange [47,48]	112-31-2	797	838	■^e^	■^d,e^	■^e^	□	□	■^e^
Undecanal	Floral, citrus, green, fresh [47]	112-44-7	847	901	■^e^	□	□	□	□	□
Dodecanal *	Citrus, green, floral [47]	112-54-9	839	922	■^e^	■^e^	□	□	■^e^	■^e^
Benzaldehyde *	Almond, burnt sugar, tropical fruit [47,48]	100-52-7	689	875	■^e^	■^e^	■^d,e^	■^e^	■^e^	■^e^
α-methylbutanal	Cocoa, almond, malt, fermented [47,48]	96-17-3	683	836	□	■^d,e^	□	□	□	■^d,e^
β-methylbutanal	Chocolate, peach [48]	590-86-3	742	861	□	□	□	□	□	■^d,e^
Phenylethanal	Honey, sweet, floral, fermented [47,48]	122-78-1	741	880	■^e^	■^e^	■^e^	□	□	■^e^
Safranal	Herb, sweet, fresh, spicy [47,48]	116-26-7	762	882	□	■^d,e^	■^e^	□	□	□
(E)-cinnamaldehyde	Sweet, cinnamon, balsamic, honey [47,48]	14371-10-9	767	868	□	■^e^	□	□	□	□
Vanillin *	Sweet, vanilla, creamy, chocolate [47,48]	121-33-5	652	721	■^e^	□	□	□	■^e^	□
**Acids**										
Acetic acid *	Acidic, sour, pungent, vinegar [47,48]	64-19-7	589	600	■^e^	■^e^	■^d,e^	■^d,e^	□	■^d,e^
Hexanoic acid	Sour, fatty, sweat, cheesy [47,48]	142-62-1	817	848	□	■^e^	■^d,e^	□	□	□
Heptanoic acid	Rancid, sour, cheesy, sweat [48]	111-14-8	785	750	□	■^e^	□	□	□	□
Octanoic acid	Acid, sweat, cheese, fruity [47,48]	124-07-2	789	837	■^e^	■^d,e^	■^e^	■^e^	□	■^e^
Nonanoic acid	Green, cheese, fatty [47,48]	112-05-0	800	830	■^e^	■^d,e^	■^d,e^	■^d,e^	□	■^e^
Decanoic acid	Rancid, fatty, sour, citrus [47,48]	334-48-5	773	814	■^d,e^	■^e^	■^d,e^	■^d,e^	■^d,e^	■^d,e^
Isovaleric acid	Sweet, acid, fermented, berry [47,48]	503-74-2	792	859	□	■^d,e^	■^d,e^	□	□	■^d,e^
Isobutyric acid	Acidic, sour, cheese, rancid [47,48]	79-31-2	754	870	■^d,e^	■^d,e^	■^d,e^	■^d,e^	□	□
Methylbutyric acid	Fruity, cheese, sweat [47,48]	759-05-7	787	839	□	□	■^d,e^	□	□	□
**Alcohols**										
1,2-epoxylinalool	Floral, alcohol [47,48]	14049-11-7	755	767	■^e^	■^e^	■^e^	■^e^	□	□
Phenylethylalcohol *	Honey, spice, rose, lilac, floral, fresh [47,48]	60-12-8	740	849	■^e^	■^e^	■^e^	■^e^	■^e^	■^e^
3-methylpentanol	Pungent, green, fruity [47,48]	589-35-5	790	919	■^e^	■^e^	□	□	□	□
Ethylhexanol	Citrus, fresh, floral, rose, green [47,48]	104-76-7	819	906	■^e^	■^e^	■^d,e^	■^e^	■^e^	■^e^
Benzyl alcohol	Floral, rose, balsamic, sweet [47,48]	100-51-6	830	855	■^e^	■^e^	□	□	■^e^	■^e^
Lauryl alcohol	Fatty, waxy, honey, coconut [47,48]	112-53-8	797	929	□	■^e^	■^e^	□	□	□
**Esters**										
Methyl salicylate	Peppermint [47], wintergreen [48]	119-36-8	690	865	■^d,e^	■^d,e^	□	■^d,e^	■^e^	□
Ethyl salicylate	Wintergreen, mint, floral, spicy [47,48]	118-61-6	792	955	■^e^	■^e^	■^d,e^	□	■^e^	■^d,e^
Methyl octanoate	Orange, vegetable, herbal [47,48]	111-11-5	632	742	□	□	■^e^	□	□	□
Ethyl octanoate	Fruity, banana, pear [47,48]	106-32-1	690	854	□	■^e^	■^e^	□	□	□
**Esters**										
Methyl palmitate	Waxy, fatty, candle [48]	112-39-0	851	888	□	□	□	□	□	■^e^
Ethyl palmitate	Fruity, milky, balsamic [48]	628-97-7	573	635	■^e^	□	■^e^	■^e^	□	□
Benzyl acetate	Fresh, boiled vegetable, fruity, floral [47,48]	140-11-4	622	722	□	■^e^	□	□	□	□
Isopropyl myristate	Faint, oily, fatty [48]	110-27-0	714	782	□	□	□	□	□	■^d,e^
**Terpenoid**										
Linalool *	Citrus, floral, lavender, sweet, green [47,48]	78-70-6	788	838	■^e^	■^e^	□	■^d,e^	■^e^	■^e^
α-terpineol	Oil, anise, mint, lemon, citrus [47,48]	98-55-5	730	838	□	□	□	■^e^	□	□
Menthol	Fresh, peppermint [47,48]	2216-51-5	761	801	□	□	□	□	□	■^e^
β-ionone *	Sweet, violet, floral, raspberry [47]	14901-07-6	708	807	□	■^e^	□	□	□	□
**Ketones**										
β-damascenone *	Apple, rose, honey, sweet, tobacco [47,48]	23726-93-4	823	927	■^e^	■^d,e^	■^e^	□	□	□
γ-nonalactone	Coconut, peach, sweet [47,48]	104-61-0	827	899	■^d,e^	■^d,e^	■^e^	■^d,e^	■^e^	□
Geranyl acetone	Magnolia, fruity, rose, pear, guava [47,48]	3796-70-1	758	873	■^e^	■^e^	■^e^	□	□	□
**Furans**										
Furfural	Bread, almond, sweet, woody [47,48]	98-01-1	867	902	■^d,e^	■^d,e^	■^d,e^	■^d,e^	■^d,e^	■^d,e^
5-methylfurfural	Almond, caramel, burnt sugar [47]	620-02-0	696	863	□	□	□	■^d,e^	□	□
2-acetylfuran	Balsamic, almond, nutty, toasted [47,48]	1192-62-7	670	848	□	□	□	■^e^	□	□
**Pyrroles**										
2-acetylpyrrole	Nutty, walnut, bread [47]	1072-83-9	680	835	□	□	□	■^e^	■^d,e^	□
Formyl pyrrole	Chocolate [48]	1003-29-8	657	819	□	■^e^	□	□	□	□
**Pyrazines**										
2,3,5-trimethyl-pyrazine	Roast, potato, musty, nutty, cocoa [47,48]	14667-55-1	689	865	□	■^e^	□	□	□	□
2,6-dimethyl-pyrazine	Nutty, butter, cocoa, caramel [47,48]	5910-89-4	695	845	□	■^e^	□	□	□	□

Note: Samples: 1—Brazil (F); 2—Bolivia (F); 3—El Salvador (F); 4—Nicaragua (F); 5—Brazil (UF); 6—Nicaragua (UF). F—fermented; UF—unfermented. * Impact compounds according to Wang et al. [24]; Xiao et al. [49]; González-Mas et al. [50]; Márquez et al. [51]; Yang et al. [52]; Dongmo et al. [53]; Schieberle and Schuh [54]. Odor description according toFlavornet [47]; and The Good Scents Company Information System [48]; ^a^ CAS# (Chemical Abstracts Service) Registry Number, available in the NIST database [24]; ^b^ELRI: Experimental Linear Retention Index; ^c^LRI: Linear Retention Index based on literature and NIST database [24]; ^d^Compounds identified with probability more than 50%; ^e^Compounds that provided a match factor higher than 600 and a match factor versus reversed match factor ratio greater than 0.8.

**Table 4:** Assessor’scharacteristics.

Gender			Age				
Male	Female		18–24	25–34	34–44	45–59	
26%	74%		39%	47%	8%	6%	
**Level of education**							
**Basic education**	**Complete high school**		**Incomplete** **graduation**		**Complete** **graduation**	**Master’s or** **Doctoral degree**	
6%	10%		31%		7%	46%	
**Family income (MW: minimum wages)**							
**1 MW**		**2–3 MW**		**4–5 MW**		**>5 MW**	
1%		25%		25%		46%	
**Frequency of tea/herbal tea consumption**				**Portionsize**			
**Daily**	**Twicedaily**	**Weekly**		**50 mL**	**Regular cup (150 mL)**	**Largecup** **(240 mL)**	
30%	16%	54%		12%	33%	55%	
**Types of teas commonly consumed**							
**Black** **tea**	**Green** **tea**	**White** **Tea**	**Toasted maté**	**Chamomile**	**Lemonbalm**	**Fruity**	Other teas *
35%	27%	6%	57%	65%	50%	44%	28%
**Bulk orsachet?**				**Brands**			
**Only** **sachets**	Only bulk teas **	**Sachetand bulk teas**		**Only** **traditional**	**Only** **imported**	**Traditionaland** **imported**	
48%	7%	45%		82%	6%	12%	
**Drinking temperature**				**Consumption of ready-to-drink** infusions ***		**Habit of** **sweetening tea**	
**Only hot**	**Only cold**	**Hot and cold**		**Yes**	**No**	Yes ****	**No**
51%	4%	45%		53%	47%	45%	55%

* Other infusions such as hibiscus, mint, bilberry, and horsetail. ** Consuming only bulk teas purchased in natural food stores or even from their gardens. *** Traditionally served cold and sweetened. **** Among those who sweetened, 77% reported using sugar and 23% sweeteners.

## Data Availability

The data presented in this study are available on request from the corresponding author.
